# Perceptions of Prominent Animal Welfare and Veterinary Care Organizations in the United States

**DOI:** 10.3390/ani10030472

**Published:** 2020-03-12

**Authors:** Mario Ortez, Courtney Bir, Nicole Olynk Widmar, Christopher A. Wolf

**Affiliations:** 1 Department of Agricultural Economics, Purdue University, West Lafayette, IN 47907, USA; nwidmar@purdue.edu; 2 Department of Agricultural Economics, Oklahoma State University, Stillwater, OK 74078, USA; courtney.bir@okstate.edu; 3 Charles H. Dyson School of Applied Economics and Management, Cornell University, Ithaca, NY 14853, USA; caw364@cornell.edu

**Keywords:** pet welfare, pet health care, best–worst, preferences

## Abstract

**Simple Summary:**

Public perception of animal well-being, and pet animal well-being in particular, remains a significant point of contention. This study ranks prominent animal welfare and veterinary care organizations’ perceived impact on pet animal well-being and health care based on U.S. residents’ perceptions, while explicitly accounting for variation between pet-owning and non-pet-owning households. Results suggest that the American Society for the Prevention of Cruelty to Animals (ASPCA) is perceived as the most impactful organization followed by the Humane Society of the United States (HSUS). The American Veterinary Medical Association (AVMA) and the American Humane Association (AHA) were tied for third most impactful. The American Animal Hospital Association (AAHA) and the American Pet Products Association (APPA) were tied for fourth most impactful while Banfield was perceived as the least impactful.

**Abstract:**

U.S. residents’ perceptions of the impact of prominent animal welfare and veterinary care organizations on pet animal well-being and health care may not be linked to the organization’s stated mission and effectiveness in advancing it, but to the level of recognition people have for the groups. An online survey of 1000 U.S. residents was used to understand the perceived impact of organizations with self-stated dedication to pet animal well-being. Using a Likert-scale, respondents ranked 13 prominent organizations as having a low to high impact on pet animal well-being and health care. The American Society for the Prevention of Cruelty to Animals (ASPCA) had the highest perceived average impact, while People for the Ethical Treatment of Animals (PETA) had the lowest. A best–worst scaling (BWS) choice experiment was conducted with 7 of the initial 13 organizations to elicit relative rankings by forcing tradeoffs by respondents. Consistent with the Likert-scale results, the ASPCA was ranked as the most impactful organization. The ASPCA’s perceived impact on pet animal well-being and health care may be linked to their high level of recognition among respondents, as this was the organization that respondents most frequently reported having seen/heard stories related to animal well-being and health care.

## 1. Introduction

Public perceptions of animal well-being and health care are the focus of increasing debate. U.S. residents can access information on animal well-being and health care from different sources: word of mouth, television, veterinarians, the Internet and, more specifically and increasingly, popular social media. In recent decades, concerns for the quality of life of animals has increasingly become the subject of public policy and controversy [1]. The rate of pet ownership in the United States was estimated at 57% at year-end for 2016 [2]. In the case of pet animals, the increase in concern is likely connected to the increase in identification of pets as much more than just companions; for example, as family members [3]. For animal agriculture, the treatment of farm animals is a central concern for livestock and poultry producers, not an isolated activist debate anymore [4]. U.S. residents’ concern for animal welfare gave rise to different non-profit and for-profit organizations focused on animal well-being and health care for pet animals and animals raised for food (i.e., livestock and poultry industries). Animal well-being-focused organizations might share similar stated goals, but each have their own agendas, structure and vision. Previous studies have questioned whether people utilize different organizations as information sources because they are more trusted than others or because they are more easily accessible or recognizable than others [5].

Some evidence suggests that over 50% of Americans do not have a source of animal welfare information or simply avoid this type of information [6,7]. The remaining segment of the population cited the HSUS and PETA as the organizations they most commonly obtain animal welfare information from [6]. Social media may be used as a way to disseminate information. Mercy for Animal (MFA) members use online and social media platforms to promote animal welfare stories [8] and PETA posted over 500 social media posts in 2016 [9]. PETA is also reported to email about 2,000,000 individuals per week with the objective of furthering their cause [10].

This analysis summarizes pet owners’ use of veterinary services and overall pet care practices. The primary objective of this study was to quantify perceptions of the U.S. public regarding prominent animal welfare and veterinary/health organizations, while acknowledging and accounting for the likely variation between pet-owning and non-pet-owning households. Past work [11] focused on evaluating and ranking the perceived (corporate) social responsibility of prominent animal welfare organizations. This work seeks to bring together prominent animal welfare organizations with those directly impacting the health of animals, namely key veterinary medicine groups. Rather than investigate the social responsibility of organizations, this analysis sought to rank which groups the U.S. public felt had the most/least impact on pet animal well-being and health. 

## 2. Materials and Methods 

### 2.1. Survey Instrument

An online survey instrument, approved by the university’s Institutional Review Board (IRB), was designed to elicit information related to public perceptions of veterinary medical services along with self-reported experience with those services. The survey was administered from July 16th until July 28th 2019 using Qualtrics, an online survey tool, to accumulate household demographic information, perceptions of health and animal well-being of different prominent animal welfare organizations, and self-reported familiarity with veterinary medical practices and procedures by U.S. residents. A largely respected company that hosts a large opt-in panel database, Kantar, was used to obtain survey respondents. Respondents were required to be 18 years of age or older to participate. The authors were not involved in the solicitation of the panel members themselves. Using quotas, the sample was targeted to be representative of the U.S. population in terms of gender, income, education, and geographical region of residence [12]. Regions of residence were defined as in the Census Bureau Regions and Division [12].

All respondents were asked questions regarding their familiarity with various prominent animal welfare, health care, and health product providing organizations. Thirteen organization were initially studied: the American Humane Association (AHA), the American Pet Products Association (APPA), the American Society for the Prevention of Cruelty to Animals (ASPCA), the American Veterinary Medical Association (AVMA), Banfield, the Human Society of the United States (HSUS), Compassion Over Killing, Mercy for Animals (MFA), People for the Ethical Treatment of Animals (PETA), Chewy.com, 1-800-PetMeds, the North American Veterinary Community (NAVC) and Veterinary Centers of America (VCA) Animal Hospitals. The questions surrounding these 13 organizations included whether respondents had heard of the organizations, whether they think they are impactful on the well-being of animals and whether they support them. Additionally, respondents were shown each organization’s logo (Table A1) and asked to drag each logo to the statement that fit best among the following options: I recognize and believe it impacts, I recognize and believe it doesn’t impact, or I have not heard of this organization. Dog and/or cat owners were asked additional questions about the acquisition method of their pets, their pet health care frequency, and their familiarity and/or experience with various veterinary services.

### 2.2. Likert Scale on Organization’s Level of Impact on Pet Animal Well-Being and Health Care

Respondents were shown a Likert-scale question for each of the 13 organizations and were asked to indicate their perceived level of impact the organization had on pet animal well-being and health care. The scale ranged from 1 (Has a low impact on pet animal well-being and health care) to 5 (Has a high impact on pet animal well-being and health care). 

### 2.3. Best–Worst Scaling (BWS) to Estimate an Organization’s Relative Perceived Impact on Pet Animal Well-Being and Health Care

While Likert-scale questions enable respondents to express their agreement in levels, BWS forces respondents to make tradeoffs that more closely reflect reality. For example, while a respondent can rank multiple factors as important in a Likert-scale question, the BWS experiment forces choices amongst those factors. In-depth assessment of relative ranking among organizations was performed through a BWS choice experiment, where respondents were asked to indicate the “greatest impact” and “least impact” on pet animal well-being and health care for different groupings of 7 of the initial 13 prominent animal health and well-bring related organizations. The 7 organizations selected for inclusion in the BWS experiment included the top four organizations with largest preference shares in Widmar et al. [11], with three added organizations that are more closely tied to pet health care. The organizations included in the BWS experiment were: the American Humane Association (AHA), the American Pet Products Association (APPA), the American Society for the Prevention of Cruelty to Animals (ASPCA), the American Veterinary Medical Association (AVMA), Banfield, the American Animal Hospital Association (AAHA) and the Human Society of the United States (HSUS). All respondents participated in the BWS experiment regardless of whether they owned a pet or not at the time of the survey. 

Within the BWS experiment, respondents were asked to select the organization that had the “greatest impact” and “least impact” on pet health and well-being from the presented subset of three organizations. This question was asked seven times, with different combinations of three of the organizations as answer options so in aggregate respondent’s perception of the different organizations could be assessed. The combination of organizations and overall design was determined by maximizing D-efficiency in SAS [13]. The location of the respondent’s perception of an organization’s impact between greatest impact to lowest impact is represented by *λ_j_*. The level of impact of an organization *j* on animal well-being and health perceived by respondent *i,* which is unobservable to the researcher, is given by:

$$I_{ij}=\lambda_{j}+\varepsilon_{ij}$$

where *ε_ij_* denotes a random error term. The probability that the respondent *i* chooses organization *j* as the organization with the greatest impact, and organization *k* as the one with the lowest impact, is the probability that the difference between *I_ij_* and *I_ik_* is greater than all potential differences available for the options shown to each participant. Assuming that the error term is the independently and identically distributed type I extreme value, the probability of selecting a greatest impact or lowest impact combination takes the multinomial logit form [14] represented by:

$$Prob\;\left(j=greatest\;impact\cap k=lowest\;impact\right)=\frac{e^{\lambda_{j}-\lambda_{k}}}{\sum_{l=1}^{J}\sum_{m=1}^{J}e^{\lambda_{l}-\lambda_{m}}-J}.$$

The parameter *λ_j_* was estimated using the maximum likelihood estimation (MLE). It represents how impactful an organization is relative to the least impactful organization. One organization must be normalized to zero to prevent multicollinearity [14].

A multinomial logit model, which allows for homogeneity among individuals, was estimated. A Random Parameters Logit (RPL) was also estimated to allow for continuous heterogeneity among individuals [14]. Both models were estimated using NLOGIT 6.0. The individual parameter estimates from the RPL model are not directly intuitive to interpret, and so they were used to calculate individual preference shares to facilitate interpretation [14]. The shares of preferences are calculated as:

$$Share\left(j\right)=\frac{e^{\lambda_{j}}}{\sum_{k=1}^{J}e^{\lambda_{k}}}.$$

The shares must sum to one across the seven organizations. The preference share for each organization is the forecasted probability that each organization is chosen as the most impactful [15].

The Latent Class Model (LCM) classifies individuals into one of *s* classes based on their attitudes, preferences and demographics. Preferences are heterogeneous across classes but homogeneous within each class [16]. The parameters for each class are simultaneously estimated and each respondent is assigned to an unobserved latent class [17]. Given that the respondent belongs to a specific latent class *s*, the conditional probability of choices is represented as:

$$Prob\;\left(j=greatest\;impact\cap k=lowest\;impact|s\right)=\frac{e^{\lambda_{js}-\lambda_{ks}}}{\sum_{l=1}^{J}\sum_{m=1}^{J}e^{\lambda_{ls}-\lambda_{ms}}}.$$

The parameters *λ_js_* and *λ_ks_* are class specific [18]. The probability that an individual participant is in any given unobservable latent class takes the multinomial logit form:

$$Prob\left(s\right)=\frac{e^{\theta_{s}Z}}{\sum_{s=1}^{S}e^{\theta_{s}Zk}}$$

where *Z* is a set of hypothesized drivers of class membership, the *s^th^* parameter vector is normalized to zero for model identification, and *θ_s_* characterizes the impact the driver has on class membership [18]. 

The make up of the classes in terms of each respondent is probabilistic, and this model allows for the estimation of the probability that an individual respondent is in any of the latent classes. The preferences within each latent class are homogeneous. The difference between the highest and the next highest probability class was calculated for each individual [19]. If the difference between their 1st and 2nd highest probability classes is small, the overall make up of each class may be skewed by respondents with a lower probability of being a member of that particular class [19]. To ensure that this classification method was robust, individuals who had less than a 50% difference between their highest probability class assignment and the next highest probabilistic class assignment were dropped from the post-estimation analysis. The demographic results of each class were summarized and statistically compared using the test of proportions.

## 3. Results

A total of 1000 respondents completed the survey and the sample demographics closely mirrored the targeted demographics with a few statistically different exceptions (Table 1). There were lower percentages of respondents in the sample who were 18–24 years (10%), did not graduate from high school (5%) and from the Midwest (21%) when compared to the U.S. Census targets. There were higher percentages of respondents who attended college (30%) and from the South (38%) when compared to the U.S. Census target of 27% and 21%, respectively.

Respondent’s pet ownership status including type and number of pets is summarized in Table 2. Fifty-nine percent of respondents indicated that they owned a pet at the time they took the survey; eight percent did not currently have a pet but had one in the past five years, and five percent said they plan to acquire a pet in the next five years. Seventy-two percent of pet-owning respondents currently own at least one dog and 56% of pet-owning respondents currently own at least one cat.

Further questions were asked to respondents who indicated that they currently own or have owned in the past five years at least one dog (n = 477) or one cat (n = 352) and their answers were statistically compared in Table 3. Eighty-seven percent of both dog and cat owners indicated that they are the primary care provider for their pet. Adopted or rescued was the most common acquisition method for dog owners (53%) and cat owners (69%). Although a statistically higher percentage of cat owners adopted or rescued when compared to dogs. Among pet owners, 35% purchased their dog and 16% purchased their cat, which is statistically different between the two species.

Forty-four percent of dog owners regularly walk or exercise their dog, compared to a statistically lower percentage of cat owners (17%). A statistically higher percentage of dog owners (50%) indicated that they have an annual veterinary visit for preventive health compared to 41% for cat owners. Although over half of dog and cat owners used a veterinarian of any kind, a statistically higher percentage of dog owners (65%) visited a veterinarian when compared to cat owners (58%). Additionally, 21% of dog and cat owners reported they used a low-cost spay neuter or low-cost vaccination clinic (24% of dog owners and 21% of cat owners). Seven percent of cat owners indicated that they never seek veterinary care, compared to only 5% of dog owners, but these numbers were not statistically different. A statistically higher percentage of dog owners (37%) indicated that they seek veterinary care more than once a year compared to only 18% of cat owners.

Dog and cat owners were asked questions regarding how they would classify their veterinarian and the affiliation of the clinic they most commonly frequented (Table 4). Most of the pet owners indicated that they visit a local independent clinic/veterinarian (70% dogs and 67% cat owners). Fewer pet owners indicated that they visit a chain clinic/veterinarian (10% of dog and 9% of cat owners).

Table 5 shows that a high percentage of respondents indicated that they have heard of PETA but chose not to support them (42%). The group that pet owners indicated to support the most regularly was Chewy (9%). Over 60% of pet owners indicated that they have not heard of MFA, Compassion Over Killing, the APPA and the NAVC.

Table 6 summarizes the responses from the logo drag-and-drop questions by pet owner and non-pet owner. The ASPCA was indicated by the highest percentage of pet and non-pet owners (72% pet owners and 62% non-pet owners) as both a recognized and impactful organization for the well-being and health of animals. PETA was selected as recognized but not impactful for the well-being and health of animals by the highest percentage of pet and non-pet owners (28% pet owners and 22% non-pet). The NAVC was the organization with the highest percentage of pet owners and non-pet owners indicating that they have not heard of the organization (59% of pet owners and 79% of non-pet owners).

**Figure 1:**
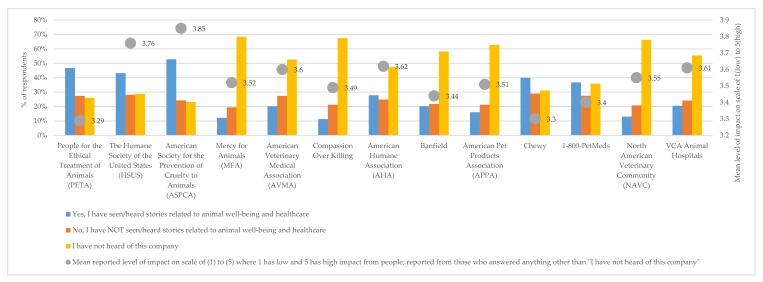
Respondents reported knowledge of animal well-being and health care organization and their mean reported level of impact (For % of respondents n = 1000); for mean level of impact, only those who answered anything other than “I have not heard of this company” were shown the rank question and included in the mean calculation).

Figure 1 compares the mean responses on a Likert scale to the question “I have seen/heard stories related to animal well-being and health care for this organization.” Generally, organizations with higher percentages of respondents indicating that they have not heard of it also had lower scores on the impact level on animal well-being and health care by respondents who indicated anything other than “I have not heard of this company”. An exception to this was PETA, where almost 50% of respondents indicated that they heard/saw stories related to animal well-being and health care, but the mean reported level of impact was low—in fact, the lowest across the organizations.

The mean preference shares from the RPL model indicate that respondents perceive the ASPCA as the most impactful organization on animal well-being and health care, as shown in Table 7. The mean preference share was 38% for the ASPCA, and it was statistically higher than the preference share for the HSUS (20%), the second largest preference share. The mean preference shares for the AVMA and the AHA were not statistically different than one another, thus both are tied for third most impactful organization on pet animal health and well-being. Additionally, there was a tie between the AAHA and the APPA for fourth. Banfield’s preference share was 5%, which is statistically lower than that for the APPA and the AAHA (6% and 9% respectively).

**Figure 2:**
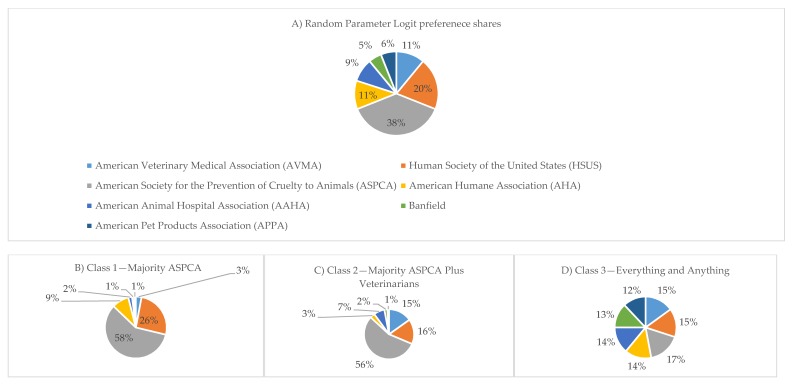
Preference shares of animal welfare organizations determined by (**A**) Random Parameters Logit and (**B**–**D**) Latent Class Model (n = 1000).

In addition to evaluating the RPL results, which present the mean preference share across all respondents, the LCM was estimated to discern mean preference shares amongst classes, or groups, of respondents (Table 8). The LCM model with three classes, which are heterogeneous across classes, but homogeneous within classes, was selected using Akaike Information Criterion (AIC) and Bayesian Information Criterion (BIC). Class 1 was dubbed “Majority ASPCA” due to the mean preference share for the ASPCA dominating at 58%, the HSUS having 26%, and all other preference shares being below 10%. Class 2 was dubbed “Majority ASPCA Plus Veterinarians” due to its heavy preference for the ASPCA and the HSUS, just as in Class 1, but with 15% allocated to the AVMA. Class 3 was dubbed “Everything and Anything” because the preference shares for every organization evaluated were roughly equal, ranging from the 12% for APPA to 17% for the ASPCA (Figure 2).

Eight hundred and forty-seven respondents were assigned to a specific latent class using the criteria of the highest probability of class membership being at least 50% over the next highest probability. The remaining 153 respondents were not assigned to a class, as their difference in class membership probability fell short of the 50% criteria. A summary of the demographics of each class from the LCM is provided in Table 9.

There were statistical differences in the demographics of respondents across latent classes. Class 1 was statistically different than class 3 with respect to gender, age, income for all but $0–$2,4999, and the West region. Class 2 was statistically different than class 3 for gender, age (except for the 35–44 years old), income (only $2,5000–$49,999 and $10,0000 and higher), education (only graduated from high school, did not attend college and Associates or Bachelor’s Degree earned). Class 1 was not statistically different than class 2 with the exception of education (graduated from high school, did not attend college and Associates or Bachelor’s Degree earned).

## 4. Discussion

At the time of the survey, 59% of respondents owned at least one pet, nearly identical to the 2016 year-end 57% reported in the AVMA’s Pet Ownership and Demographics Sourcebook 2017–2018 [2]. One aspect of cat ownership is semi-ownership, where people care for cats by providing food and shelter, but do not ‘own’ the cat [22]. People with semi-ownership believe that cats are more independent [22], which may result in decreased veterinary care and less recognition of pain in cats or injury in cats [23]. The bond that people perceive they have with their cat or dog impacts veterinary care, with pets that have a closer bond receiving more veterinary care. Since cats tend to be more aloof, they tend to receive less veterinary care. However, cat owners tend to be more educated than dog owners, which presents a unique opportunity for veterinarians to extend care [24]. Given the complex pain masking biology of cats, and the unique bond people have with dogs, understanding differences between cat and dog owners was of importance in this manuscript.

The organizations which respondents indicated that they have not heard of or they have not seen/heard stories regarding animal well-being and health care the most were MFA and Compassion Over Killing. This is consistent with a related study where out of seven prominent animal welfare organizations, MFA and Compassion Over Killing had the fewest respondents indicating that they have heard/seen animal welfare stories from [11]. Respondents indicated that they have seen/heard stories related to animal well-being and health care the most from the ASPCA followed by PETA. The same two organizations were positioned at the top of the list in the social responsibility study [11]. Given the notable public information and awareness campaigns that both the ASPCA and PETA run, and the scale of their budgets for such, including television commercials and national/international media, the recognition of these organizations is unsurprising.

Public perception of organizations focused on animal well-being may be the result of a combination of marketing campaigns including TV ads and social media presence. Meanwhile, the actual impact they have on the pet population may be unknown by the average U.S. resident. This study finds that generally, organizations that U.S. residents haven seen/heard stories related to pet animal well-being and health care also have a higher reported level of impact in this regard. PETA was an exception to this finding. Likert-scale questions results revealed that the ASPCA had the highest perceived average impact consistent with the BWS experiment results in this study. While respondents self-reported their perceptions about impact of organizations, it was not investigated whether the impact was perceived to be exclusively positive. Given the verbiage employed in the survey instrument, it is possible that a respondent assigned high impact to an organization believing that impact was good, but it was also possible that high impact was tied with negative outcomes. In other words, it is possible to be impactful in a positive way or in a negative way and the directionality of impact was beyond the scope of data collected in this study. In a related study, the ASPCA was found as the most trusted source of breeding-dog welfare information when compared with other scientific, veterinary and industry sources and organizations [5]. The results suggest that the ASPCA’s perceived level of impact on pet animal welfare and well-being is accompanied with a high level of trust when it comes to other pet animal topics such as breeding-dog welfare. Past evidence suggests that PETA and the HSUS are the most commonly cited source of animal welfare information in the U.S. [6]. This is likely linked to their high level of activity in advertising and communication via media campaigns on issues pertaining to livestock and poultry health care [25]. In this analysis, PETA had the lowest average perceived impact on animal well-being and health care. Together, these results suggest that while PETA is a common source of animal welfare information and is very active in communications, it is not perceived as a highly impactful organization pertaining to pet animal health care and well-being. The HSUS was perceived to have the second highest impact on pet animal well-being and welfare, which might suggest that their popularity as a source of animal welfare information is combined with a perceived high level of impact on this topic.

The RPL results suggest that respondents perceive the ASPCA as the most impactful organization on animal well-being and health care, having the highest preference share. This is consistent with Bir et al. [5] where the ASPCA was found to be the most trusted source of breeding-dog welfare, when compared with other scientific, veterinary and industry sources and organizations [5]. It is also consistent with Widmar et al. [11], where the ASPCA was found to be perceived as the most socially responsible organization, and in both studies, the ASPCA had the highest preference share. The HSUS had the second highest preference share in this study, consistent with Widmar et al. [11], where the HSUS also had the second highest preference share. Based on estimated preference shares from the BWS choice experiment, the AVMA was tied with the AHA for third most impactful organization in this study. Widmar et al. [11] found the AVMA as fourth for most socially responsible and Bir et al. [5] found the AVMA as third most trusted source of breeding-dog welfare.

Three classes were identified from the LCM in this study. Respondents in class 1 showed a strong affinity for the ASPCA, representing 58% of the preference, similar to Bir et al. [5] where one of thier LCM classes showed a strong preference for the ASPCA (69.8%). The overall evidence in this study (BWS choice experiment, Likert scale questions, and general perceptions questions) suggests that the ASPCA is perceived as the most impactful organization on animal well-being and health care. This perceived impact level is likely influenced by the ASPCA’s high visibility and respondent’s familiarity as reported within the study. However, high visibility does not universally imply high impact; PETA had a high level of visibility, but respondents reported a low perceived level of impact on animal well-being and health care.

## 5. Conclusions

This study found that public perception of prominent animal welfare and veterinary care organizations among U.S. residents might be linked to the organization’s level of visibility. In general, organizations that had higher level of visibility also had a higher reported level of impact. PETA, for which a high percentage of respondents indicated to have seen/heard stories related to animal well-being and healthcare but with the lowest reported level of impact, was an exception to the general finding. ASPCA was perceived as the most effective organization and it was also the organization that respondents most frequently reported having seen/heard stories related to animal well-being and healthcare. 

Organizations may wish to know how they rank in terms of perceived impact against other groups within the public realm. Individuals seeking to make contributions or provide support for animal health care or well-being organizations may judge organizations based on their perceived impact relative to organizations with similar missions. Thus, while it is recognized that public perception does not imply or reflect effectiveness, it may reflect popularity and/or visibility.

Admittedly, the interpretability of the relative ranking of the organizations studied is limited by the experimental design employed and the organizations included in the experiment. This analysis sheds light on the public perceptions of diverse organizations on pet animal health and well-being. Inclusion of alternative organizations may alter the relative ranking and preference shares, by nature of the experiment. Thus, the interpretability and ranking order are specific to the experiment conducted. Future research may wish to investigate public perceptions of impact on specific species of animals or to delve into groups positive versus negative impacts on well-being.

## Figures and Tables

**Table 1:** Survey demographics (n = 1000), percent (%) of respondents.

Demographic Variable	Respondents	U.S. Census
Gender		
Male	49	49
Age		
18–24	10 ^†^	13
25–34	18	18
35–44	18	16
45–54	19	17
55–65	17	17
65+	19	19
Income		
$0–$24,999	22	22
$25,000–$49,999	23	23
$50,000–$74,999	17	17
$75,000-$99,999	11	12
$100,000 and higher	26	26
Education		
Did not graduate from high school	5 ^†^	13
Graduated from high school; did not attend college	30	28
Attended College; no Degree earned	23	21
Attended College; Associates or Bachelor’s Degree earned	30 ^†^	27
Attended College; Graduate or Professional Degree earned	13	12
Region		
Northeast	18	18
South	38 ^†^	21
Midwest	21 ^†^	38
West	24	24

^†^ Percentage of respondents is statistically different than the percentage of the U.S. census.

**Table 2:** Pet ownership statistics (n = 1000), percent (%) of respondents.

Pet Ownership	
Currently have pet animal(s)	59
Do not currently have pet animal(s) but have in the past 5 years	8
Plan to acquire pet animal(s) in the next 5 years	5
None	29
**Type of pets that respondents currently have, percentage of pet owners who have at least one n = 588**	
Dog	72
Cat	56
Fish	12
Horse	4
Bird	8
Reptile	5
Rabbit	3
Small Mammal ^1^	5
Other	4
**Type of pets that respondents that do not currently have a pet but had in the past 5 years had, percentage of pet owners who had at least one n = 76**	
Dog	70
Cat	33
Fish	13
Horse	5
Bird	7
Reptile	1
Rabbit	5
Small Mammal ^1^	7
Other	3

^1^ Small mammal includes a hamster, ferret, guinea pig, rat, mouse, chinchilla, or gerbil.

**Table 3:** Pet-owning respondent pet care and usage of veterinary services. Percent (%) of dog- (n = 477) or cat- (n = 352) owning respondents.

Statement	Dogs	Cats
**How have you acquired your dog(s)/cat(s))? ^1^**		
Purchased	35 ^θ^	16
Adopted or rescued	53 ^θ^	69
Received as a gift	19	18
Other	6	8
**Action taken regarding dog(s)/cat(s) health ^1^**		
Regularly (5–7x/week) walk or exercise	44 ^θ^	17
Has an annual veterinary visit for preventative health	50 ^θ^	41
Subscribes to or follows on social media veterinary health experts or sources	11	9
Has participated in formal obedience classes with the dog(s)	14	
Has visited with a behavioral specialist	9	7
None of the above	15 ^θ^	38
**Types of practices/service providers used for dog(s)/cat(s) ^1^**		
Low-cost spay/neuter clinic	21	21
Low-cost vaccination clinic	24	21
Veterinarian/Clinic/Practice of any kind	65 ^θ^	58
Emergency veterinary clinic	16 ^θ^	9
Ambulatory veterinary services (i.e., in-home care)	6	5
Veterinary college provided services	10 ^θ^	5
Veterinary surgery center	13 ^θ^	8
Specialty veterinary service center/clinic (e.g., allergy testing, ophthalmologist, etc.)	8	8
Other	3 ^θ^	9
**Frequency of seeking veterinary care for your dog(s)/cat(s)**		
Never	5	7
Only in emergencies	14 ^θ^	29
Once a year	41	42
More than once a year	37 ^θ^	18
I don’t know	3	4

^θ^ Percentage of dog-owning respondents is statistically different than the percentage of cat-owning respondents at 5% significance level. ^1^ Multiple selections permitted.

**Table 4:** Pet owner-reported veterinarian classification and vet clinic affiliation, percent (%) of dog (n = 477) and cat (n = 352) owners.

Statement	Dogs	Cats
**Veterinary Classification**		
Local independent clinic/veterinarian	70	67
Nationally affiliated (chain) clinic/veterinarian	10	9
Mobile pop-up clinic/veterinarian	7	5
Cat clinic only	NA	6
Clinic/veterinarian affiliated with veterinary college	12	8
Other	1 ^θ^	4
**The veterinary clinic I most often frequent is affiliated with ^1^**		
Banfield Pet Hospitals^®^	12	10
Veterinary Centers of America (VCA)	15	11
BluePearl	7	7
Pet Partners	11	7
None of the above	35	38
I don’t know	37	42

^θ^ Percentage of dog-owning respondents is statistically different than the percentage of cat-owning respondents at the 5% significance level. ^1^ Multiple selections permitted.

**Table 5:** Summary of respondent’s knowledge and support of prominent organizations, percent (%) of dog (n = 477) and cat (n = 352) owners.

Organization	I Have NOT Heard of This Group	I Have Heard of This Group but Do NOT Support Them Because Opportunities to Are Not Easily Accessible to Me	I Have Heard of This Group but Do NOT Support Them Because I Choose Not to	I Support This Group Only Occasionally	I Support This Group Regularly
People for the Ethical Treatment of Animals (PETA)	25	16	42	11	6
The Humane Society of the United States (HSUS)	32	17	28	15	8
American Society for the Prevention of Cruelty to Animals (ASPCA)	24	19	30	18	8
Mercy for Animals (MFA)	66	11	13	6	5
American Veterinary Medical Association (AVMA)	57	13	19	7	5
Compassion Over Killing	66	10	12	6	5
American Humane Association (AHA)	48	13	22	10	7
Banfield	58	12	17	7	6
American Pet Products Association (APPA)	61	13	15	6	5
Chewy	34	19	27	10	9
1-800-PetMeds	37	18	31	9	6
North American Veterinary Community (NAVC)	65	10	14	6	6
VCA Animal Hospitals	57	12	18	7	6
Total	48	14	22	9	6

**Table 6:** Recognition and perceived impact of organizations, percent (%) of pet owners (n = 664) and non-pet owners (n = 336).

Organization ^a^	I Recognize and Believe It Impacts		I Recognize and Believe It Doesn’t Impact		I Have Not Heard	
	Pet Owner	No Pet	Pet Owner	No Pet	Pet Owner	No Pet
American Humane Association (AHA)	4 ^θ^	34	12	10	40 ^θ^	56
American Pet Products Association (APPA)	32 ^θ^	15	12	8	56 ^θ^	78
American Society for the Prevention of Cruelty to Animals (ASPCA)	72 ^θ^	62	14	13	14 ^θ^	26
American Veterinary Medical Association (AVMA)	33 ^θ^	15	10	8	57 ^θ^	78
Banfield	38 ^θ^	16	15 ^θ^	8	47 ^θ^	76
Compassion over Killing	31^θ^	14	13 ^θ^	6	56 ^θ^	81
The Humane Society of the United States (HSUS)	70 ^θ^	54	13	10	17 ^θ^	36
Mercy for Animals (MFA)	32 ^θ^	16	11 ^θ^	6	58 ^θ^	77
People for the Ethical Treatment of Animals (PETA)	53 ^θ^	45	28	22	19 ^θ^	33
Chewy.com	54 ^θ^	29	24	20	21 ^θ^	51
1-800-PetMeds	56 ^θ^	31	23	18	21 ^θ^	51
North American Veterinary Community (NAVC)	31 ^θ^	11	11	9	59 ^θ^	79
VCA Animal Hospitals	41 ^θ^	20	10	7	49 ^θ^	74

^θ^ Percentage of dog-owning respondents is statistically different than the percentage of cat-owning respondents at the 5% significance level. ^a^ Pet owners assigned organizations to three possible statements by dragging and dropping logos of organizations into the specified ‘buckets’ of “I recognize this organization and believe it impacts the well-being and health of pet animals”, “I recognize this organization and do not believe it impacts the well-being and health of pet animals” or “I have not heard of this organization”.

**Table 7:** Multinomial logit (MNL) and random parameter logit (RPL) results (n = 1000).

Model	MNL	RPL			
Organization	Coefficient (St Err)	Coefficient (St Err)	Standard Deviation (St Err)	Shares of Preferences (Confidence Interval)	Rank
American Veterinary Medical Association (AVMA)	0.5587 ***	0.7044 ***	0.5415 ***	11	3
	(0.0316)	(0.0398)	(0.0528)	[0.1063, 0.1239]	
Human Society of the United States (HSUS)	0.9077 ***	1.2761 ***	1.1413 ***	20	2
	(0.0327)	(0.0537)	(0.0607)	[0.1881, 0.2215]	
American Society for the Prevention of Cruelty to Animals (ASPCA)	1.2174 ***	1.8991 ***	1.6776 ***	38	1
	(0.0345)	(0.0732)	(0.0784)	[0.3465, 0.4128]	
American Humane Association (AHA)	0.5206 ***	0.6617 ***	0.6798 ***	11	3
	(0.0316)	(0.0416)	(0.0524)	[0.1017, 0.1191]	
American Animal Hospital Association (AAHA)	0.3609 ***	0.4349 ***	0.0529	9	4
	(0.0315)	(0.0349)	(0.0784)	[0.0815, 0.0942]	
Banfield	−0.0191	−0.1642 ***	1.0005 ***	5	5
	(0.0320)	(0.0490)	(0.0584)	[0.0435, 0.0532]	
American Pet Products Association (APPA)	-	-	-	6	4
	-	-	-	[0.0524, 0.0612]	

*** Indicates significance at the 0.001 level; ^1^ The rank was determined by examining overlapping confidence intervals established using the Krinsky–Robb method [20]. Examination of overlapping confidence intervals is intuitive and allows visual comparison when confidence intervals are presented. Comparing of (95%) confidence intervals for overlap is more conservative than the standard method of significance testing when the null hypothesis is true and falsely fails to reject the null hypothesis more frequently than the standard method when the null hypothesis is false [21].

**Table 8:** Latent Class Model results.

Classes	Class 1		Class 2		Class 3	
Organization	Coefficient (St Err)	Shares of Preferences	Coefficient (St Err)	Shares of Preferences	Coefficient (St Err)	Shares of Preferences
American Veterinary Medical Association (AVMA)	1.2775 ***	3	1.9599 ***	15	0.1726 ***	15
	(0.1236)		(0.2886)		(0.0479)	
Human Society of the United States (HSUS)	3.4702 ***	26	2.0206 ***	16	0.1799 ***	15
	(0.2517)		(0.2332)		(0.0489)	
American Society for the Prevention of Cruelty to Animals (ASPCA)	4.3070 ***	59	3.2494 ***	55	0.3254 ***	17
	(0.2949)		(0.2862)		(0.0551)	
American Humane Association (AHA)	2.4144 ***	9	0.3250 **	3	0.1331 ***	14
	(0.2748)		(0.1449)		(0.0459)	
American Animal Hospital Association (AAHA)	0.7180 ***	2	1.1934 ***	7	0.1369 ***	14
	(0.0952)		(0.2040)		(0.0449)	
Banfield	−0.390 ***	1	−0.1329	2	0.0966 **	13
	(0.0997)		(0.1815)		(0.0464)	
American Pet Products Association (APPA)	-	1	-	1	-	12
	-		-		-	
Probability of class membership	0.3101 ***		0.1795 ***		0.5105 ***	

*** Indicates significance at the 0.001 level; ** significance at the 0.05 level.

**Table 9:** Comparison of Latent Class Model class demographics and class assignment methods.

Demographic	Percent (%) of Respondents Assigned to Highest Probability Class, Only Respondents with a 50% Difference in Highest and Next Highest Probability (n = 847)		
	Class 1 (n = 270)	Class 2 (n = 111)	Class 3 (n = 466)
Gender			
Male	42 ^b^	40 ^c^	56
Age			
18–24	5 ^b^	5 ^c^	14
25–34	11 ^a,b^	7 ^c^	25
35–44	10 ^b^	11 ^c^	25
45–54	21 ^b^	23	17
55–65	24 ^b^	20 ^c^	10
65+	28 ^a,b^	35 ^c^	9
Income			
$0–$2,4999	19	19	23
$2,5000–$4,9999	20 ^b^	18 ^c^	27
$5,0000–$7,4999	19 ^b^	20	14
$7,5000–$9,9999	10 ^b^	8	14
$10,0000 and higher	31 ^b^	35 ^c^	22
Education			
Did not graduate from high school	1 ^b^	2	6
Graduated from high school; did not attend college	30 ^a^	23 ^c^	33
Attended College; no Degree earned	24	22	21
Attended College, Associates or Bachelor’s Degree earned	30 ^a^	42 ^c^	27
Attended College; Graduate or Professional Degree earned	14	11	13
Region			
Northeast	18	20	19
South	40	39	36
Midwest	22	23	18
West	20 ^b^	19	27
Pet owner	57	56	62

^a^ Percent of respondents in Class 1 is statistically different than the percentage of respondents in Class 2 at the 0.05 level; ^b^ Percent of respondents in Class 1 is statistically different than the percentage of respondents in Class 3 at the 0.05 level; ^c^ Percent of respondents in Class 2 is statistically different than the percentage of respondents in Class 3 at the 0.05 level.

## Appendix A

**Table A1:** Organization’s logos.

Organization Name	Organization Logo
American Humane Association (AHA)	
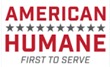
American Pet Products Association (APPA)	
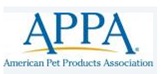
American Society for the Prevention of Cruelty to Animals (ASPCA)	
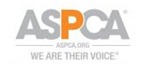
American Veterinary Medical Association (AVMA)	
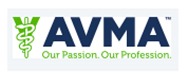
Banfield	
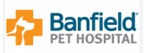
Compassion over Killing	
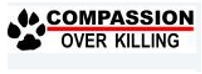
The Humane Society of the United States	
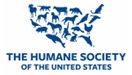
Mercy for Animals	
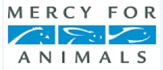
PETA	
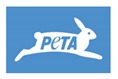
Chewy.com	
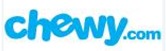
1-800-PetMeds	
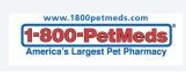
NAVC	
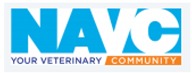
VCA Animal Hospitals	
